# Multimodal Classification of Schizophrenia Patients with MEG and fMRI Data Using Static and Dynamic Connectivity Measures

**DOI:** 10.3389/fnins.2016.00466

**Published:** 2016-10-19

**Authors:** Mustafa S. Cetin, Jon M. Houck, Barnaly Rashid, Oktay Agacoglu, Julia M. Stephen, Jing Sui, Jose Canive, Andy Mayer, Cheryl Aine, Juan R. Bustillo, Vince D. Calhoun

**Affiliations:** ^1^The Mind Research Network and Lovelace Biomedical and Environmental Research InstituteAlbuquerque, NM, USA; ^2^Psychology Department, University of New MexicoAlbuquerque, NM, USA; ^3^Department of Electrical and Computer Engineering, University of New MexicoAlbuquerque, NM, USA; ^4^Psychiatry Department, University of New Mexico School of MedicineAlbuquerque, NM, USA; ^5^Psychiatry Research Program, New Mexico VA Health Care SystemAlbuquerque, NM, USA; ^6^Department of Neurosciences, University of New Mexico School of MedicineAlbuquerque, NM, USA; ^7^Neurology Department, University of New Mexico School of MedicineAlbuquerque, NM, USA; ^8^Department of Radiology, University of New Mexico School of MedicineAlbuquerque, NM, USA

**Keywords:** connectivity, fMRI, MEG, Schizophrenia, static and dynamic functional connectivity, classification

## Abstract

Mental disorders like schizophrenia are currently diagnosed by physicians/psychiatrists through clinical assessment and their evaluation of patient's self-reported experiences as the illness emerges. There is great interest in identifying biological markers of prognosis at the onset of illness, rather than relying on the evolution of symptoms across time. Functional network connectivity, which indicates a subject's overall level of “synchronicity” of activity between brain regions, demonstrates promise in providing individual subject predictive power. Many previous studies reported functional connectivity changes during resting-state using only functional magnetic resonance imaging (fMRI). Nevertheless, exclusive reliance on fMRI to generate such networks may limit the inference of the underlying dysfunctional connectivity, which is hypothesized to be a factor in patient symptoms, as fMRI measures connectivity via hemodynamics. Therefore, combination of connectivity assessments using fMRI and magnetoencephalography (MEG), which more directly measures neuronal activity, may provide improved classification of schizophrenia than either modality alone. Moreover, recent evidence indicates that metrics of dynamic connectivity may also be critical for understanding pathology in schizophrenia. In this work, we propose a new framework for extraction of important disease related features and classification of patients with schizophrenia based on using both fMRI and MEG to investigate functional network components in the resting state. Results of this study show that the integration of fMRI and MEG provides important information that captures fundamental characteristics of functional network connectivity in schizophrenia and is helpful for prediction of schizophrenia patient group membership. Combined fMRI/MEG methods, using static functional network connectivity analyses, improved classification accuracy relative to use of fMRI or MEG methods alone (by 15 and 12.45%, respectively), while combined fMRI/MEG methods using dynamic functional network connectivity analyses improved classification up to 5.12% relative to use of fMRI alone and up to 17.21% relative to use of MEG alone.

## Introduction

While brain function is highly complex at the microscopic scale, brain imaging data itself is also high dimensional and complex. High dimensional, complex data challenge our ability to identify patterns using standard observational approaches whereas sophisticated classification algorithms may more easily reveal patterns that improve patient classification accuracy. Functional brain imaging provides an opportunity to assess brain function noninvasively and new knowledge is being gained as additional data mining methods are developed. In addition to providing insights into normal functioning of the healthy brain, new approaches are needed to further elucidate into complex mental illnesses such as schizophrenia which is a heterogeneous disorder characterized by positive and negative symptoms as well as cognitive impairments. Research studies have identified gray and white matter abnormalities and disrupted connectivity across large-scale brain networks in schizophrenia (Mohamed et al., 1999; Kubicki et al., 2007). Such dysconnectivity may be driven by aberrant synaptic plasticity (Stephan et al., 2009) though the underlying mechanisms of the disorder are still unclear. Characterizing functional connectivity provides an opportunity to help understand schizophrenia at the macro scale and may help us better understand how various brain regions are impacted.

The majority of previous classification studies have primarily focused on a single neuroimaging method such as functional magnetic resonance imaging (fMRI) or magnetoencephalography (MEG). It has been shown that exclusive reliance on a single method has some limitations (Calhoun and Sui, 2016). Also multiple previous studies used very small numbers of subjects for training classifiers combined with high-dimensional datasets limiting the ability to design robust and accurate classifiers for schizophrenia.

To overcome the limitations imposed by exclusive reliance on a single method and by small training datasets, some studies have increased the variety of data types. In a recent study (Silva et al., 2014), researchers were asked to automatically diagnose schizophrenia using both structural and functional MRI data. The results showed that investigators who used both MRI data types achieved better accuracy relative to those who used only one. Similarly, previous work (Sui et al., 2011) aiming to highlight differences between schizophrenia and bipolar disorder by combining fMRI and diffusion tensor imaging (DTI) data, linked function–structure networks by investigating the joint components with strong links between DTI and fMRI. They achieved higher estimation accuracy than by using a single imaging modality. The combination of multiple data types has also been extended to three modalities in the application of multi-set canonical correlation analysis to combine data of resting state fMRI and EEG with structural MRI (sMRI) (Sui et al., 2014). Results showed that ensemble features from these methods improve the classification accuracy between patients and controls significantly. Similarly, Dai et al. (2012) introduced an automatic classification framework for attention deficit/hyperactivity disorder by combining fMRI and sMRI. Results of their experiments showed that using multimodal features from fMRI and sMRI yielded better classification results for attention deficit/hyperactivity disorder patients. Another study (Cetin et al., 2015a) used a sensory loaded task hierarchy to increase the data variety and pointed out the importance of multitask information (in this case sensory loading) to discover effective features to classify schizophrenia patients (SZs) and healthy controls (HCs). Wang et al. (2012) examined the spatial concordance between MEG and fMRI for a verb generation task. MEG and fMRI data showed spatial convergence in the same anatomical regions. Also, Ingalhalikar et al. (2014) aimed to classify autism spectrum disorder using MEG based auditory tasks and fractional anisotropy and diffusivity measures from DTI. They also achieved higher accuracy then single modality classifiers. Bridwell and Calhoun (2014) performed a review of studies using fMRI or EEG or the combination of both. The conclusions of this review as well as other work on the topic have quite consistently demonstrated that the combination of EEG-fMRI networks show combining information from multiple modalities provides an improved ability to isolate brain networks, and may help clarify their potentially distinct roles in assessing cognition and behavior when using EEG or fMRI alone.

Previous studies showed that increasing the variety of data types helps researchers to gain a broader understanding of mental disorders. Specifically, MEG and fMRI which are two well-known neuroimaging approaches have become very popular in recent years. fMRI and MEG provide complementary information, both of which provide a vast amount of data that are not easily modeled or summarized without the loss of potentially critical information. For instance; whilst the blood oxygenation-level dependent (BOLD) response measured by fMRI allows better visualization of the extent or distribution of the activated area, it is limited by measuring an indirect and slow physiological signal (Kim et al., 1997). On the other hand, neural oscillatory activity, which comprises rhythmic electrical activity in cell assemblies, is thought to underlie BOLD responses. Neural oscillations measured with MEG occur in the ~1–900 Hz band (Cohen, 1968; Xiang et al., 2009); such rapid electrical signals cannot be directly assessed using fMRI. Therefore, using both fMRI and MEG, within a common sample of subjects, would combine the strengths of each modality by allowing comparison of both hemodynamic and electrophysiological effects.

Significant progress toward integrating fMRI and MEG has been made in the past decade. A recent study (Brookes et al., 2011a) estimated intrinsic connectivity networks (ICNs) in MEG in a similar way to that typically used in fMRI (that is extracting coherent patterns from source space data; Calhoun et al., 2001). Also, our recent study (Houck et al., in press) used a method based on spatial group independent component analysis (Sg-ICA), for the first time estimating functional network connectivity (FNC) from both MEG and fMRI. Spatial group independent components resulting from Sg-ICA are maximally spatially independent but their corresponding time-courses can provide a significant amount of temporal dependency. ICA based FNC is a data-driven approach that measures the functional connectivity (cross-correlation) among brain functional networks (Jafri et al., 2008; Çetin et al., 2014). The results suggest that the combination of these two methods provides complementary information that captures essential characteristics of functional network connectivity.

The ability to extract important disease related features from MEG and fMRI imaging data is an extremely important topic. We used a Sg-ICA approach (Erhardt et al., 2011a; Calhoun and Adali, 2012), excluded artifactual brain networks (Jafri et al., 2008) from fMRI and MEG methods by using FNC, and then conducted a classification study of schizophrenia which is a common psychiatric condition characterized by gray and white matter abnormalities and disrupted connectivity across large-scale brain networks. Such dysconnectivity includes disruption of both structural (Kubicki et al., 2007) and FNC (Calhoun et al., 2008; Jafri et al., 2008; Çetin et al., 2014; Rashid et al., 2015; Houck et al., in press) that may be related to clinical symptoms, including cognitive dysfunction.

Previous studies showed that classification accuracy between patients and controls is improved by combining data from different modalities or incorporating dynamic data. Using multimodal data and a dynamic analysis pipeline may provide us with unique disease related features. There have been a few classification paper which have shown the promise of such data (Silva et al., 2014; Arbabshirani et al., 2016). However, to our knowledge no work looking directly at MEG and fMRI in the context of both static and dynamic connectivity.

In this study, we used the same resting state data set analyzed in Houck et al. (in press). Resting state data is particularly advantageous for the study of disease states where patients may have difficulty responding or performing behavioral tasks due to compromised cognitive and/or physiological function. Also, we recruited a relatively large number of subjects (91) with almost equal distribution across disease group (47 SZ, and 44 HC). Hence this data set well suited for a classification analysis.

Different than the previous study, which described the group level differences in networks obtained with Sg-ICA using both MEG and fMRI, this study now uses this large fMRI/MEG data set to examine the usefulness of combining fMRI and MEG for performing single-subject classification. We hypothesized that the combination of fMRI and MEG modalities would identify unique trait-based information that can be used for individual prediction of mental illness such as schizophrenia at the single-subject level. Also we used static (traditional approach) and dynamic (newer approach) FNC pipelines to show that improvement of classification accuracy is not dependent on a specific FNC pipeline. The purpose of the present study was to use both fMRI and band limited envelope correlation metrics from MEG to interrogate static and dynamic functional connectivity in the resting state in a sample of HCs and SZs to improve the classification accuracy of SZs.

## Materials and methods

### Participants

The data used in this study were from 91 subjects (46 SPs and 45 HCs). Informed consent was obtained from all subjects according to institutional guidelines at the University of New Mexico Human Research Protections Office, and all data were anonymized prior to group analysis. All participants were compensated for their participation with cash following each study visit. All participants were between the ages of 18–65 years.

HCs were recruited from the same geographic location and completed the Structured Clinical Interview for DSM-IV Axis I Disorders—Non-Patient Edition to rule out Axis I conditions (First et al., 2002). Additional exclusion criteria for HCs included a current or past psychiatric disorder (with the exception of one lifetime depressive episode), depression or antidepressant use within the past 6 months, lifetime antidepressant use of more than 1 year, and history of a psychotic disorder in a first-degree relative. Exclusion criteria for both groups included a history of neurological disorder, head trauma with loss of consciousness >5 min, mental retardation, active substance dependence or misuse within the past year and lifetime history of dependence or use within the last 12 months of phencyclidine, amphetamines, or cocaine.

Schizophrenia patients were recruited from the University of New Mexico (UNM) Hospitals and the Albuquerque Veterans Administration Medical Center. Inclusion criteria for patients with schizophrenia included a diagnosis of schizophrenia based on the Structured Clinical Interview for DSM-IV-TR. The inclusion criteria for patients was a diagnosis of schizophrenia or schizoaffective disorder. Participants with a history of neurological disorders including head trauma (loss of consciousness >5 min), mental retardation, alcohol, drug dependence, or abuse (except for nicotine) within the past year were excluded. Each SZ patient completed the Structured Clinical Interview for DSM-IV Axis I Disorders (First et al., 2002) for diagnostic confirmation and evaluation for co-morbidities. The clinical core (COBRE Stability Clinic—Dr. Bustillo) affiliated with this project determined retrospective stability from relevant psychiatric records documenting no change in symptomatology or type/dose of psychotropic medications were recorded for these patients in the 3 months prior to either scan. The clinical core assessed prospective stability during three consecutive weekly visits and during each imaging assessment. SZ patients were considered prospectively stable if they demonstrated no change in clinical symptoms >2 points in the positive symptom items on the positive and negative syndrome scale (PANSS: Kay et al., 1987), no score of “worse” or “much worse” on the clinical global impression scale (Guy, 1976), expressed no suicidal or violent ideation, and had no psychiatric or medical hospitalizations. Doses of antipsychotic medications were converted to olanzapine equivalents (OE) (Gardner et al., 2010). We ran two-sample *t*-test between OE scores and loading factors for fMRI and all MEG frequencies. The results showed not significant effect (*p* > 0.05) of OE scores on the loading parameters. All SZ patients had a negative toxicology screen for drugs of abuse at the start of the study. All participant smokers were instructed not to use tobacco during the 2 h prior to each scan to minimize acute effects. This was confirmed via a breath carbon monoxide measure of <8 ppm. SZs and HCs were matched on parental educational level (*p* > 0.05), a less biased estimate of pre-morbid educational attainment potential (Saykin et al., 1991). We assessed symptom severity among the SZs group with the PANSS. Table 1 provides demographic characteristics of the participants. Each participant completed resting MEG and MRI scans. Each imaging session was between 1 and 2 h. Scans were collected in counterbalanced order, with a median time between scans of ~22 days.

**Table 1:** **Demographic and clinical variables for SZs and HCs**.

	**HC (*n* = 44) Mean (*SD*)**	**SZ (*n* = 47) Mean (*SD*)**	***t* or *x*^2^(*p*-value)**
**DEMOGRAPHICS**			
Age	37.28 (13.86)	35.18 (11.83)	0.78 (0.44)
Gender (M/F)	37/7	34/13	0.27 (0.78)
Ethnicity (H/NH)	23/21	26/21	
**RACE**			
American Indian/Alaska Native	2	2	
Asian	2	0	
African American	1	4	
Native Hawaiian or Other Pacific Islander	1	0	
White	38	41	
Age of onset of psychosis	20.04 (8.03)		
Illness duration	16.22 (12.91)		
Calgary depression	3.25 (1.01)		
			
**SOCIOECONOMIC STATUS**			
PCEL			
CODEM-6	4.24 (2.11)	4.53 (1.18)	0.85 (0.15)
CODEM-7	4.72 (1.83)	4.72 (1.84)	0.75 (0.35)
Nicotine	0.51 (1.24)	0.91 (1.73)	0.79 (0.2)
Motion (mean framewise displacement in mm)	0.210 (0.124)	0.275 (0.192)	1.87 (0.07)
**PANSS**			
Positive	15.13 (5.14)		
Negative	15.15 (5.01)		
General	29.79 (8.108)		
**MEDICATIONS**			
OE (mg/day)	14.02 (12.39)		

*Ethnicity: H, Hispanic; NH, Non Hispanic; PANSS, Positive and Negative Syndrome Scale. PCEL, Primary caregiver education level. CODEM-6, Highest Level of Education for Primary Caretaker until 18 years old. CODEM-7, Highest Level of Education for Secondary Caretaker until subject was 18 years old. Educational levels as follows: 1, grade 6 or less; 2, grade 7–12; 3, graduated high school; 4, part college; 5, graduated 2 year college; 6, graduated 4 year college; 7, graduate or professional school; 8, completed graduate or professional school. Calgary, Calgary Depression Scale. Nicotine, Nicotine use across groups. OE, Olanzapine Equivalence*.

### Functional MRI analysis

#### fMRI data acquisition

fMRI data were collected on a 3-Tesla Siemens Trio scanner at the Mind Research Network with a 12-channel radio frequency coil with a repetition time of 2 s. High-resolution T1-weighted structural images were acquired with a five-echo MPRAGE sequence with TE = 1.64, 3.5, 5.36, 7.22, 9.08 ms, TR = 2.53 s, TI = 1.2 s, flip angle = 7°, number of excitations = 1, slice thickness = 1 mm, number of slices = 27, field of view = 256 mm, resolution = 256 × 256. The RMS average of these echoes was used as the final T1 image. T2^*^-weighted functional images were acquired using a gradient-echo EPI sequence with TE = 29 ms, TR = 2 s, flip angle = 75°, slice thickness = 3.5 mm, slice gap = 1.05 mm, field of view 240 mm, matrix size = 64 × 64, voxel size = 3.75 × 3.75 × 4.55 mm, number of slices = 29,210 frames and ascending acquisition (Allen et al., 2011a; Mayer et al., 2012). Resting-state scans consisted of 149 volumes.

#### fMRI data preprocessing

Resting fMRI data were preprocessed using an automated preprocessing pipeline (Bockholt et al., 2009) based on the SPM toolbox (http://www.fil.ion.ucl.ac.uk/spm/) implemented in MATLAB (www.mathworks.com). The first six volumes were discarded to remove T1 equilibration effects. Next, to realign the images, the INRIalign algorithm (Freire et al., 2002) was used, and slice-timing correction was applied using the middle slice as the reference frame in the functional data pipeline. The data were then spatially normalized to the standard Montreal Neurological Institute (MNI) space (Friston et al., 1995) using a nonlinear (affine + low frequency direct cosine transform basis functions) registration, resampled to 3 × 3 × 3 mm voxels, and smoothed using a Gaussian kernel with a full-width at half-maximum (FWHM) of 10 mm. The preprocessed time series data was scaled to a mean of 100. This intensity normalization improves the test-retest reliability of the group independent component analysis (GICA; Allen et al., 2011b).

#### fMRI spatial group independent component analysis (Sg-ICA)

We used the MATLAB-based (www.mathworks.com) GIFT Toolbox (http://mialab.mrn.org/software/gift/) and infomax algorithm (Bell and Sejnowski, 1995) for Sg-ICA. The Sg-ICA approach was selected over temporal group ICA for three reasons: (1) Because components produced by Sg-ICA are not temporally independent, relations among network timecourses can be evaluated; (2) temporal group ICA of participant timecourses carries the assumption of temporal consistency, limiting its utility in group analysis of resting data; and (3) Sg-ICA is more robust to motion than seed based approaches (Damaraju et al., 2014a).

The subject-specific data reduction principal component analysis was performed and 100 principal components retained by using a standard economy-size decomposition (Allen et al., 2011b). The relatively large number of subject-specific principal components has been shown to stabilize subsequent back-reconstruction (Erhardt et al., 2011b). Then reduced data from all subjects and all sessions were concatenated together and put through another PCA reduction step. Further group data reduction was performed to use memory more efficiently by using an expectation maximization (EM) PCA algorithm (Roweis, 1998) and 75 PCs were retained.

We used a relatively high model order ICA (number of components, C = 75), since such models yield refined components that correspond to known anatomical and functional segmentation (Kiviniemi et al., 2009; Abou-Elseoud et al., 2010). In order to estimate the reliability of the decomposition, the Infomax ICA algorithm was applied repeatedly (20 times) in ICASSO (http://research.ics.aalto.fi/ica/icasso/). Best estimated runs were selected by using the quality of component clusters that reflects the difference between intra-cluster and extra-cluster similarity and resulting components were clustered (Himberg et al., 2004).

The ICA results, once estimated and fixed, can be considered as a set of weighted seed maps (Joel et al., 2011). The ICA algorithm identifies maximally statistically-independent sets of maps (which can overlap) each of which are represented by a strongly coherent (correlated) time-course. A major strength of multivariate approaches like ICA is that in the case of a distributed set of regions, there are multiple locations, which are highly correlated to one another, and thus can be considered a node in this sense. Indeed, as one knows that all the voxels with strong weights in a given component are highly correlated, and thus it makes sense to consider them a node and use FNC to evaluate the relationship among these nodes (Erhardt et al., 2011a).

#### fMRI non-artifactual components identification

**Figure 1:**
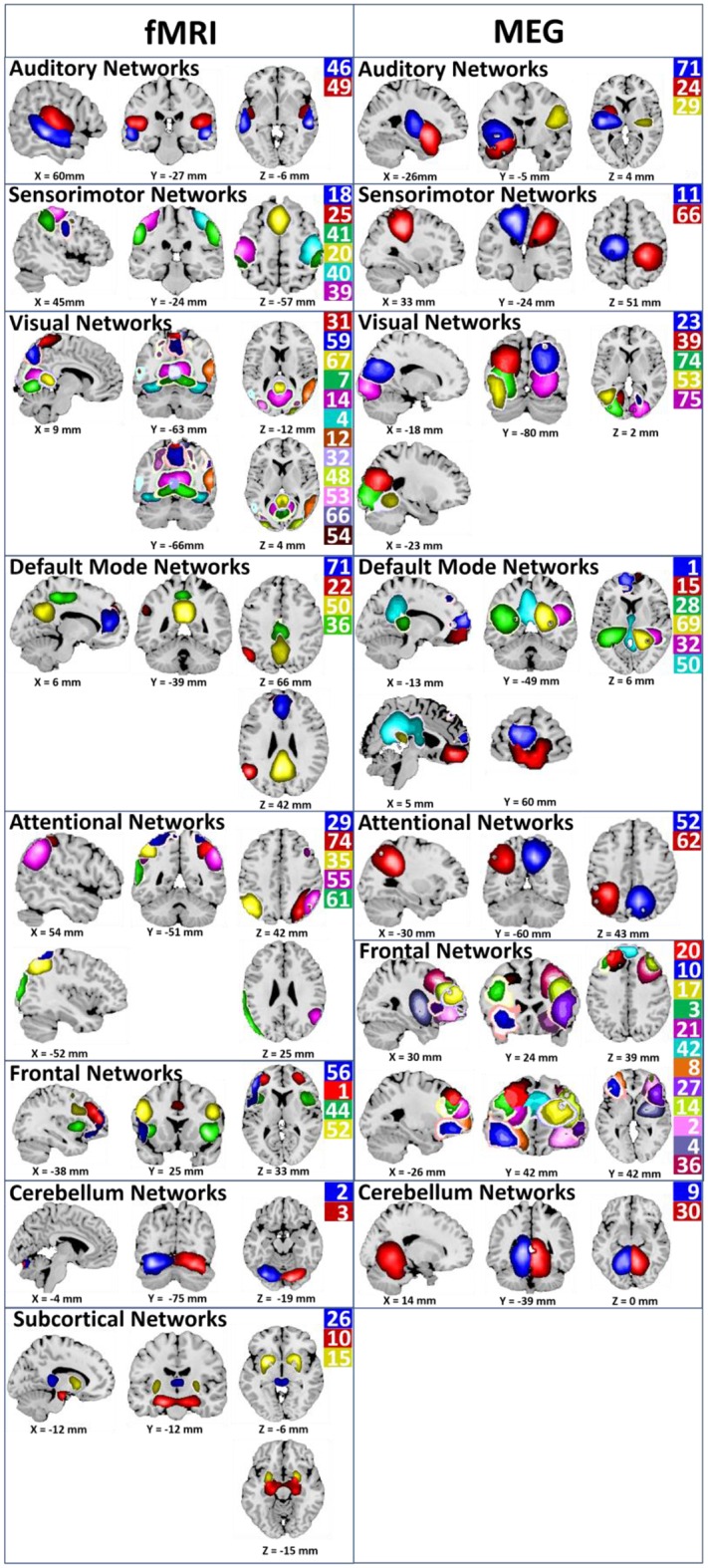
**Non-artifactual ICA components for fMRI and MEG**. Non-artifactual components are divided into groups based on their anatomical and functional properties and include auditory networks (Aud), sensory motor network (SM), visual network (Visual), default mode network (DMN), attentional network (Att_Cg), frontal network (Frontal), cerebellar network (Cer), and subcortical network (SbCor). ICA component numbers are listed for each functional networks. The same color used for component picture and component number (Houck et al., in press).

Out of the 75 components returned by the Sg-ICA, 39 were labeled (see Figure 1) as non-artifactual independent components (IC) using a combination of two methods (Allen et al., 2011a; Çetin et al., 2014) for fMRI (see Table 2). In the first method, the power spectra were examined with two criteria in mind: dynamic range and low frequency/high frequency ratio. Dynamic range refers to the difference between the peak power and minimum power at frequencies to the right of the peak in the power spectra. Low frequency to high frequency power ratio is the ratio of the integral of spectral power below 0.10 Hz to the integral of power between 0.15 and 0.25 Hz. To verify the results, reviewers evaluated the components for functional relevance. In this evaluation, the three expert reviewers investigated if a component exhibited (1) peak activation in gray matter, (2) low spatial overlap with vasculature and ventricles, (3) low motion and susceptibility artifacts, and (4) timecourses (TCs) dominated by low frequency fluctuations, it was classified as a non-artifactual component. We primarily used the peak functional region for anatomical labeling of non-artifactual ICs.

**Table 2:** **Anatomical labels (based on the peak functional region) of non-artifactual independent components from the fMRI analysis**.

**No**.	**Anatomical labels**
**fMRI COMPONENTS**	
1	Left anterior cingulate cortex
2	Right cerebellum
3	Left cerebellum
4	Middle occipital gyrus
7	Right fusiform gyrus and left cerebellum
10	Left heschl's gyrus
12	Middle temporal gyrus
14	Right lingual gyrus
15	Putamen
18	Postcentral gyrus
20	Left (SMA + Precentral gyrus)
22	Left angular gyrus
25	Right inferior frontal gyrus
26	Left thalamus
29	Left precuneus
31	Right precuneus
32	Cerebellar vermis
35	Angular gyrus
36	Middle cingulate cortex
39	Left postcentral gyrus
40	Right postcentral gyrus
41	Supramarginal gyrus
44	Insula lobe
46	Middle temporal gyrus
48	Middle occipital gyrus
49	Right heschl's gyrus + Left superior temporal gyrus
50	Left posterior cingulate cortex
52	Right precuneus + Right inferior frontal gyrus
53	Right Inferior occipital gyrus
54	Left cuneus
55	Right angular gyrus + Left inferior parietal lobule
56	Left inferior frontal gyrus
59	Right precuneus
61	Left angular gyrus + Right precuneus
66	Right precuneus + Left paracentral lobule
67	Left lingual gyrus
71	Left anterior cingulate cortex
74	Right angular gyrus

### MEG analysis

#### MEG data acquisition

MEG data were collected in a magnetically shielded room (VAC Series Ak3B, Vacuumschmelze GmbH) using a whole-cortex 306-channel MEG array (Elekta Neuromag™) at the Mind Research Network. Before positioning the participant in the MEG, four head position indicator (HPI) coils were affixed to the participant's head: two on the forehead and one behind each ear. These coils allow determination of the position of the participant's head relative to the position and orientation of the MEG sensors. Additional positioning data were collected using a head position device (Polhemus Fastrak) to permit co-localization of MEG activity with each participant's anatomical MRI. Two channels of electro-oculogram (EOG), one vertical and one horizontal, and one channel of electrocardiogram (ECG) were collected simultaneously with MEG. MEG data were sampled at a rate of 1000 Hz, with a bandpass filter of 0.10–330 Hz. Head position was monitored continuously throughout the MEG session using the four HPI coils that were energized continuously during the scan at known frequencies (293, 307, 314, and 321 Hz, respectively). Raw data were collected and stored. Participants were instructed to keep their eyes open and maintain fixation during the 6-min scan to minimize occipital alpha rhythm (Kim et al., 1997).

#### MEG data preprocessing

Artifact removal, correction for head movement, and down sampling to 250 Hz were conducted offline using Elekta Maxfilter software, with 123 basis vectors, a spatiotemporal buffer of 10 s, and a correlation limit of 0.95. To facilitate comparison with previous research (Brookes et al., 2011b), data were bandpass filtered into four frequency ranges of interest: delta (1–4 Hz), theta (5–9 Hz), alpha (10–15 Hz), beta (16–29 Hz), and gamma (30–50 Hz).

The MaxMove function of the Maxfilter software (http://imaging.mrc-cbu.cam.ac.uk/meg/Maxfilter) for MEG eliminates the confound of head motion for MEG data by transforming the MEG data to the same head position across each participant's scan using the head position information collected throughout the scan (Taulu and Kajola, 2005). Motion was detected using the correlation of spatial patterns between each time instance and the last known head position. Head position fits were estimated once per second when no motion was detected or more often when the correlation dropped below *r* = 0.98. Complete details of the method can be found elsewhere (Taulu and Kajola, 2005; Taulu and Simola, 2006).

As a part of MEG beamformer projection, covariance matrices were generated independently for each subject and frequency band, using all recorded data. Covariance matrices were regularized using a value of 4 times the minimum singular value of the unregularized matrix. Voxels were placed on a regular 6-mm grid spanning the brain image. Source orientation at each voxel was based on a nonlinear search for maximum projected signal-to-noise ratio. The forward solution was based on a dipole model (Sarvas, 1987) and a single-shell boundary element model (Hämäläinen and Sarvas, 1989). Beamformer projection was performed separately for each subject and frequency range. Then source-space signals were normalized by an estimate of projected noise (Hall et al., 2013) and transformed to standard MNI space using FLIRT in FSL. A Hilbert transform was applied to the timecourse at each voxel to derive the analytic signal. The Hilbert envelope at each voxel was down sampled to an effective sampling rate of 1 Hz (Brookes et al., 2011b). Thus, resting-state scans consisted of 300 volumes for each MEG frequency band. Source space envelope data were smoothed spatially (6 mm at full-width half-maximum), and the voxel size was resampled to 3 × 3 × 3 mm to facilitate comparison with the fMRI data.

#### MEG spatial group independent component analysis (Sg-ICA)

Sg-ICA was applied to the individual subject data using the GIFT toolbox (http://mialab.mrn.org/software/gift). Each frequency range was treated as a session in GIFT to permit analysis of each band, as well as the mean across bands.

MEG ICA processing generally followed the procedures applied to the fMRI data. Reduction steps were applied using principal component analysis. First, subject-specific data reduction was applied, retaining 100 principal components. Next, group level data reduction was applied to reduce the dataset to 75 principal components. Infomax ICA was applied 20 times in ICASSO and the resulting components were clustered. Spatial maps were generated by decomposing the mixed MEG timecourses to yield a set of spatially independent and temporally coherent networks. As with fMRI, FNC was computed as the zero-lag cross-correlations among reconstructed timecourses.

#### MEG non-artifactual components identification

For MEG ICA processing and non-artifactual components selection, we follow the procedures that are applied to the fMRI as mentioned in the previous section. Of the 75 components obtained from the group ICA, 32 were retained as non-artifactual components for MEG (see Table 3).

**Table 3:** **Anatomical labels (based on the peak functional region) of non-artifactual independent components for MEG method**.

**No**.	**Anatomical labels**
**MEG COMPONENTS**	
1	Left rectal gyrus
2	Right middle orbital gyrus
3	Left middle frontal gyrus
4	Right putamen
8	Left superior frontal gyrus
9	Right cerebellum (IV-V)
10	Left inferior frontal gyrus
11	Left paracentral lobule
14	Right inferior frontal gyrus
15	Superior medial gyrus
17	Right superior frontal gyrus
20	Left superior frontal gyrus
21	Left inferior frontal gyrus
23	Right superior occipital gyrus
24	Left temporal pole
27	Right inferior frontal gyrus
28	Left superior temporal gyrus
29	Right rolandic operculum
30	Left lingual gyrus
32	Right superior temporal gyrus
36	Right Middle Frontal Gyrus
39	Middle occipital gyrus
42	Left superior medial gyrus
50	Left posterior cingulate cortex
52	Right precuneus
53	Left middle occipital gyrus
62	Left angular gyrus
66	Right postcentral gyrus
69	Right precuneus
71	Left heschl's gyrus
74	Left inferior occipital gyrus
75	Right lingual gyrus

### Functional network connectivity analysis

The FNC information was obtained from fMRI and MEG scans performed on a set of SZs and HCs, using Sg-ICA. The Sg-ICA decomposition of the preprocessed fMRI and MEG data resulted in a set of brain maps, and corresponding timecourses. These timecourses indicated the activity level of the corresponding brain map at each point in time. The FNC features are the pair-wise correlations between these timecourses, for each subject. FNC indicates a subject's overall level of “synchronicity” between brain network components (Erhardt et al., 2011a). Also, the FNC timecourses are despiked (this is the default in GIFT) which is the largest contributor to minimize motion effects (Damaraju et al., 2014a).

**Figure 2:**
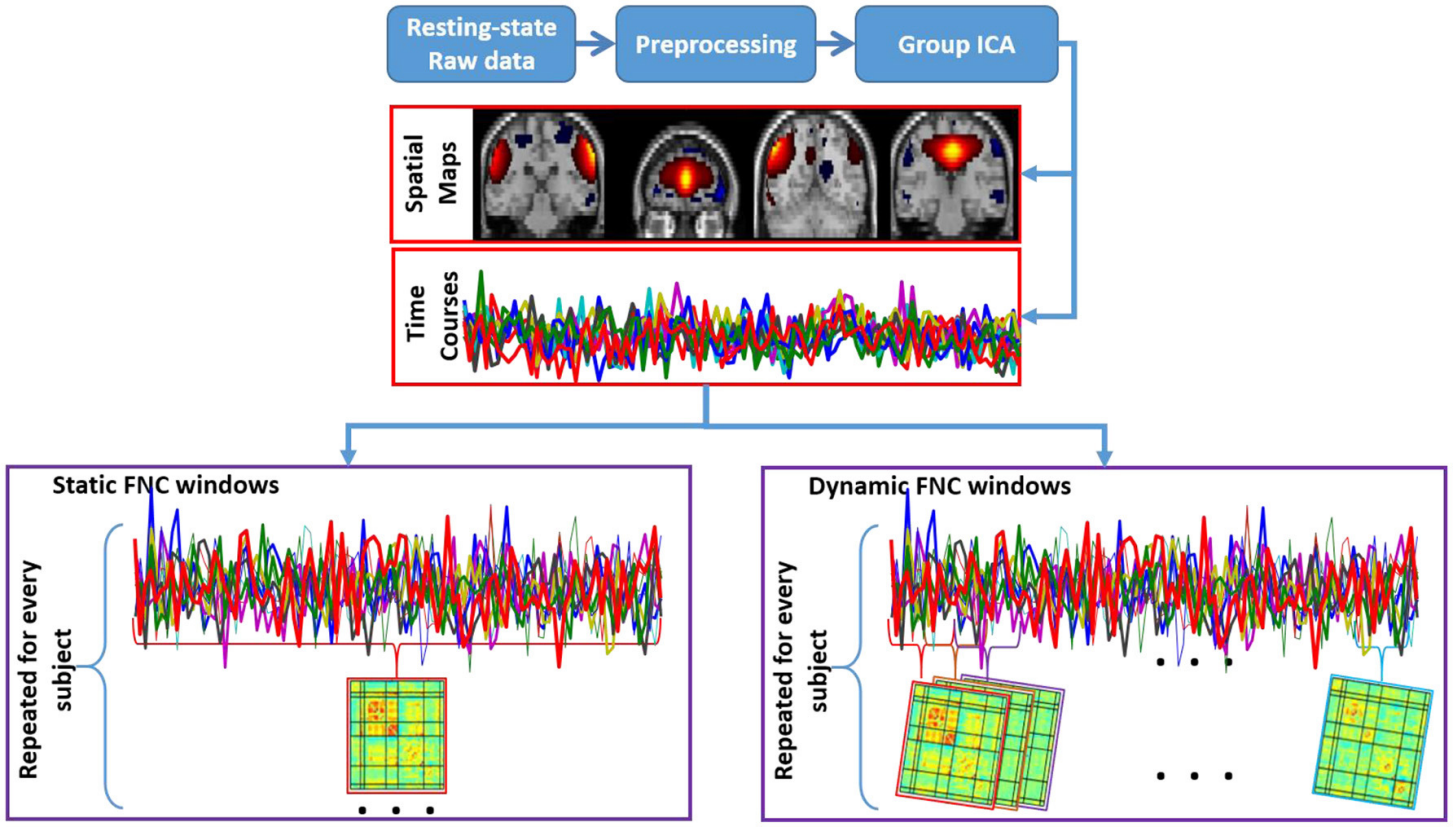
**Schematic description of static and dynamic FNC for fMRI and MEG data**.

In this study, we used static and dynamic FNC pipelines (see Figure 2). While static FNC matrices (traditional FNC pipeline) is based on correlation of full length of non-artifactual component timecourses, dynamic FNC (newer FNC pipeline) is based on smaller segment (windowed timecourses) of the correlation of non-artifactual component timecourses that captures repetitive patterns of interactions among intrinsic networks for non-artifactual component timecourses.

#### Static functional network connectivity analysis

Independent component analysis based functional connectivity is considered a high-level functional connectivity measure. *Static* functional network connectivity (*static* FNC) is a correlation value that measures the functional connectivity (cross-correlation) among brain functional networks (Jafri et al., 2008; Çetin et al., 2014) by using the full length of non-artifactual component timecourses (see Figure 2). Therefore, the *static* FNC feature gives a picture of the average connectivity pattern between independent components (average correlation matrix) for each group.

#### Dynamic functional network connectivity analysis

While static FNC is based on correlation of the full length non-artifactual component timecourses, dynamic FNC is based on correlation of windowed, non-artifactual component timecourses (see Figure 2). Recent work (Sakoğlu et al., 2010; Allen et al., 2012; Calhoun et al., 2014; Damaraju et al., 2014b; Rashid et al., 2014) suggests that connectivity dynamics can capture repetitive patterns of interactions among intrinsic networks by using a sliding window approach for non-artifactual component timecourses. These repetitive patterns of interactions during rest or task related experiments contain valuable information for individual prediction of group membership for SZs relative to HCs (Sakoğlu et al., 2010; Rashid et al., 2015). Obtaining these connectivity dynamics is named “*dynamic* functional network connectivity (*dynamic* FNC).”

### Classification and feature selection

*Static* FNC and *dynamic* FNC were used to extract reliable features for fMRI and MEG methods. We used a leave-one-out cross validation method. One subject's data was used for testing and the rest of the data (90 subjects) were used as a training data set. This process is repeated for each subject.

To examine the classification performance of the described method, three well-known classification algorithms: linear discriminant classifier (LDC), Naïve Bayes classifier (NBC), and non-linear SVM (nSVM) with Gaussian radial bases function kernel were used to test the hypothesis.

#### Static functional network connectivity and feature selection

First, we evaluated the performance of static FNC. To find most significant features, timecourse correlation of non-artifactual components were transformed to z-scores using Fisher's transformation [z = arctanh(r)] for each group and method. Then, robustness of maximum lagged correlation between each pair of timecourses was tested separately using *t*-tests. Finally, to determine the significant differences between the HCs and SZs from the training set paired *t*-tests were used. The cut-off *p*-value for all of the tests was set to *p* < 0.05 and was corrected for multiple comparisons using the false discovery rate (FDR) method (Genovese et al., 2002).

Also, we repeated the same feature selection method for fMRI and each MEG frequency band: delta, theta, alpha, beta, and gamma. Significant differences were observed between the HCs and SZs from fMRI and MEG methods. For MEG method, only delta, alpha, and beta bands showed significant group differences. Therefore, for MEG we only used static FNCs observed from these frequency bands.

#### Dynamic functional network connectivity and feature selection

**Figure 3:**
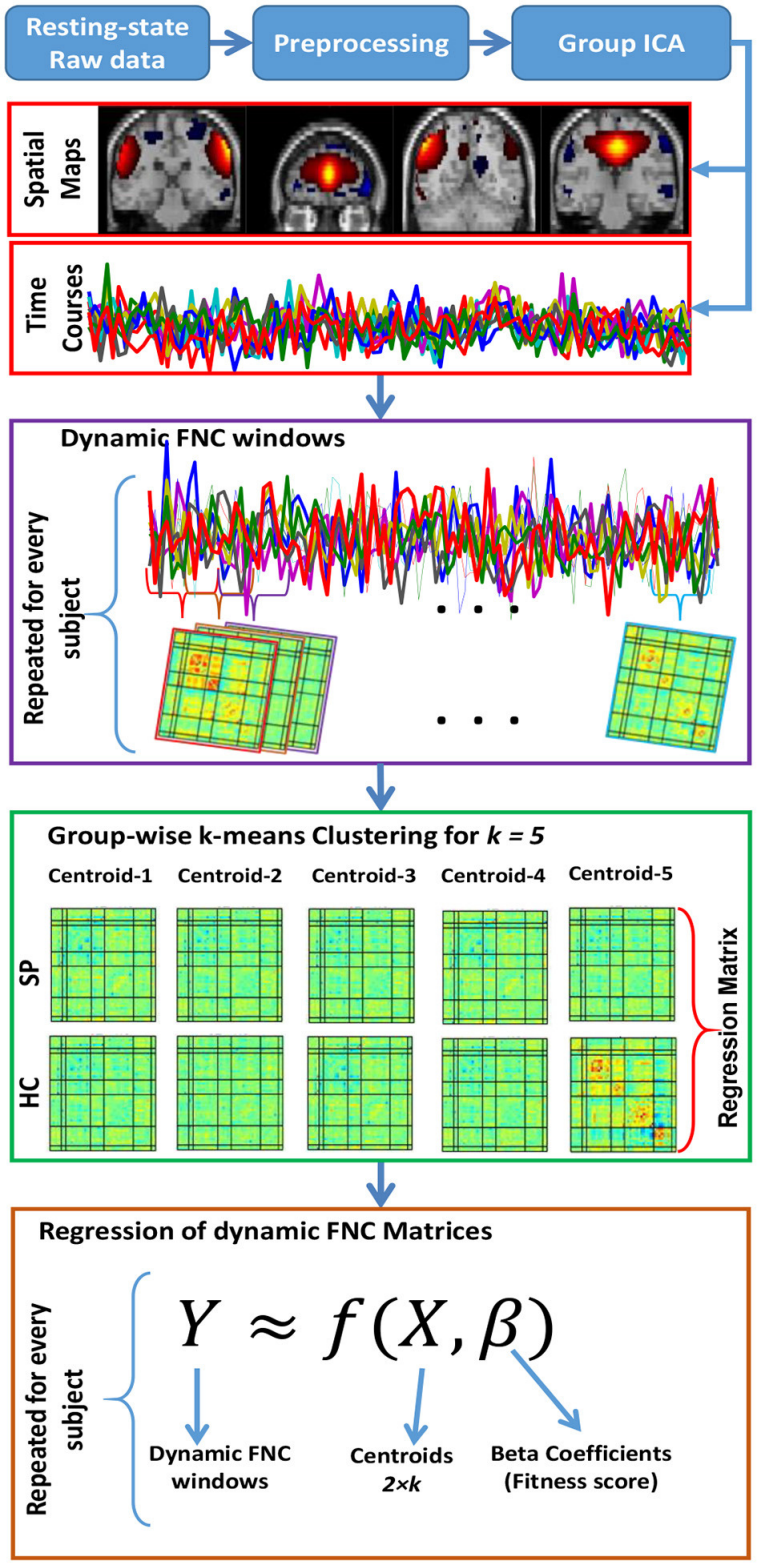
**Schematic description of dynamic FNC for fMRI and MEG data, clustering, and regression of dynamic FNC matrices**.

In order to obtain connectivity dynamics for fMRI and MEG data, (1) we computed correlations between non-artifactual component' timecourses using a sliding window approach with a default setting from GICA which is a rectangular window of 31 TR (in steps of 1TR) convolved with a Gaussian of sigma 3 TRs to obtain tapering along the edges. To characterize the full covariance matrix, we estimated covariance from the regularized inverse covariance matrix (ICOV) (Smith et al., 2011) by using the graphical LASSO framework (Friedman et al., 2008). Then we placed a penalty on the L1 norm of the precision matrix to enforce sparsity. The regularization parameter was optimized for each subject separately by evaluating the log-likelihood of unseen data of the subject in a cross-validation framework. (2) We selected group centrotypes by using k-means clustering algorithm from all of the dynamic windowed FNC matrices for each group in the training set. Then for each FNC time point, we regressed out the dynamic FNC matrix against these 2 × k states and obtained the corresponding beta coefficients. We used the mean of these beta coefficients and finalized 2 × k features. These features were used for classification of test subjects. See Figure 3 for schematic description of dynamic FNC, clustering and regression of dynamic FNC matrices.

In order to compute the optimal accuracy score, we define the most efficient cluster number by using elbow criterion of the cluster validity index, which is computed as the ratio between within-cluster distances to between-cluster distance.

In each cross-validation run, we obtained 5 cluster centroids (*k* = 5) for each group and regressed out the dynamic FNC matrix against these 10 centroids (5 centroids for each group) and computed the corresponding beta coefficients for all dynamic FNC obtained from training data set. Then, we used the mean of these beta coefficients across the subjects and finalized 10 features for classification of test subject.

To examine the combination of fMRI and MEG, the feature selection method (mentioned above) was applied to fMRI and MEG separately then features were concatenated. For each condition (fMRI, MEG, and fMRI+MEG), features were used as input data for the classification algorithms.

### Ensemble

To investigate how much improvement we achieved by combining fMRI and MEG data we used an ensemble method (Dietterich, 2000; Cetin et al., 2015b). It is used to improve the performance in classification accuracy by using the predictive power of sub samples from the original data. It has been extensively studied and has been shown to be successful in improving the performance in classification accuracy for diverse applications.

In our study, fMRI and MEG are used as sub samples of the all data. We used a simple majority voting as an ensemble method where each model in the ensemble vote has equal weight. Finally, the class that gains the majority of votes becomes the final prediction.

After we combined the features from fMRI and each MEG frequency band, we applied classification algorithms. For static FNC classification, we had three predictions that were obtained from the combination of fMRI and MEG-delta, fMRI and MEG-alpha, fMRI and MEG-beta features. Also, for dynamic FNC classification we had five predictions that were obtained from the combination of fMRI and MEG-delta, fMRI and MEG-theta, fMRI and MEG-alpha, fMRI and MEG-beta, fMRI and MEG-gamma features.

Final prediction is decided by applying the ensemble method for each FNC. Similarly, ensemble method can be used for different classification algorithms but it is not the goal of this paper.

## Results

Our main focus was to investigate the classification accuracy of fMRI, MEG and the combination of these two methods. First, the classification performance of the fMRI and MEG features was investigated individually. Then these two feature sets were combined and change in classification performance was examined. Also, the variance between the classification algorithms was reported; we used the average classification accuracy scores of these three algorithms.

### Static FNC classification

**Figure 4:**
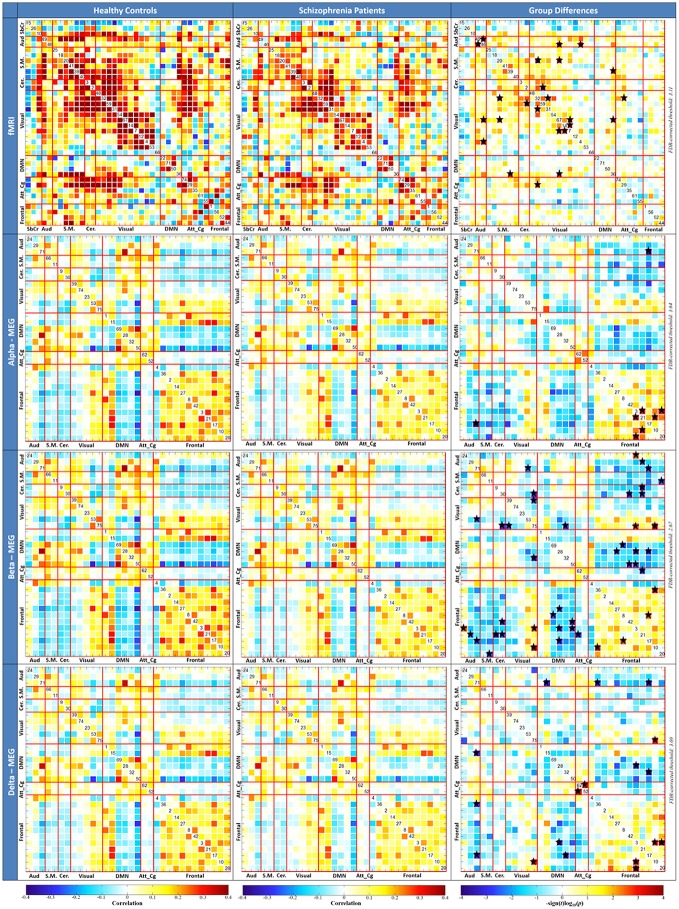
**Average static functional network connectivity (FNC) for fMRI (top) and concatenation of MEG frequencies (bottom), for HCs (left column), SZs (center column), and FDR-corrected group differences (right column)**. Significant differences between the HCs and SZs are signed with ⋆.

Static FNC results and significant differences for HCs and SZs are reported in Figure 4 for each method. These significant differences capture some interesting characteristics of brain network connectivity in patients with schizophrenia (for age range 18–65).

Seven hundred and three correlation scores for fMRI and, 496 correlation scores for MEG were obtained with *static* FNC. To determine which component pairs were significantly different between HC and SZ, two-sample *t*-tests were performed. The threshold for all of the tests is set at *p* < 0.05 and was corrected for multiple comparisons using the FDR method. The component pairs that showed significant group differences (see Figure 4) were used as features for the classification algorithms.

Two-sample *t*-tests revealed significant group differences in 12 features for fMRI, 9 features for MEG-Delta, 4 features for MEG-Alpha, 22 features for MEG-Beta, and none for theta and gamma (see Figure 4). These results point out that fMRI, MEG-Delta, MEG-Alpha, and MEG-Beta data show existence of significant difference of brain network connectivity between the HCs and SZs groups.

To compute classification accuracy scores, features were obtained from the training dataset as explained above and used for group estimation of test data. Classification accuracy scores are reported in Table 4 for fMRI, MEG-Delta, MEG-Alpha, and MEG-Beta and the combination of MEG frequencies by using ensemble method.

**Table 4:** **Classification accuracy obtained from fMRI features, MEG-Delta, MEG-Alpha and MEG-Beta features and combination of MEG features by using an ensemble method**.

**Static FNC**	**fMRI (%)**	**MEG**			
		**Delta (%)**	**Alpha (%)**	**Beta (%)**	**Ensemble (%)**
NBC	72.53	72.53	70.33	68.13	74.73
nSVM	69.23	73.63	71.43	74.73	75.82
LDF	69.23	72.53	71.43	59.34	74.73
Average	70.33	72.90	71.06	67.40	75.09
(std)	(1.91)	(0.64)	(0.64)	(7.72)	(0.63)

Ensemble results of MEG frequency bands (Delta, Alpha, and Beta) improve the classification accuracy compared to individual MEG frequency bands. Ensemble of MEG frequency bands gives the best classification accuracy score (75.09%, Average accuracy scores of classification algorithm) and outperforms fMRI. Comparison of internal MEG frequency bands for *static* FNC shows that MEG—Delta frequency (72.9%) has better performance than other frequency bands.

Table 5 reports the classification accuracy results for combined features from fMRI and all MEG frequency bands. Combination of features obtained from *static* FNC of fMRI and each MEG frequency and then these features used for each classification algorithm. Results showed that the framework with combined features provided better classification accuracy results for all methods (fMRI and all MEG frequency bands). The best performance is obtained by the combination of all by using ensemble method (85.35%).

**Table 5:** **Classification accuracy obtained from static FNC for the combination of fMRI features and MEG features for alpha, beta, and delta frequency bands and the combination of all by using ensemble method**.

**Static FNC**	**fMRI**			**Ensemble (%)**
	**MEG—Delta (%)**	**MEG—Alpha (%)**	**MEG—Beta (%)**	
NBC	82.42	80.22	75.82	85.71
nSVM	83.52	82.42	70.33	85.71
LDF	79.12	80.22	70.33	84.62
Average	81.69	80.95	72.16	85.35
(std)	(2.29)	(1.27)	(3.17)	(0.63)

**Figure 5:**
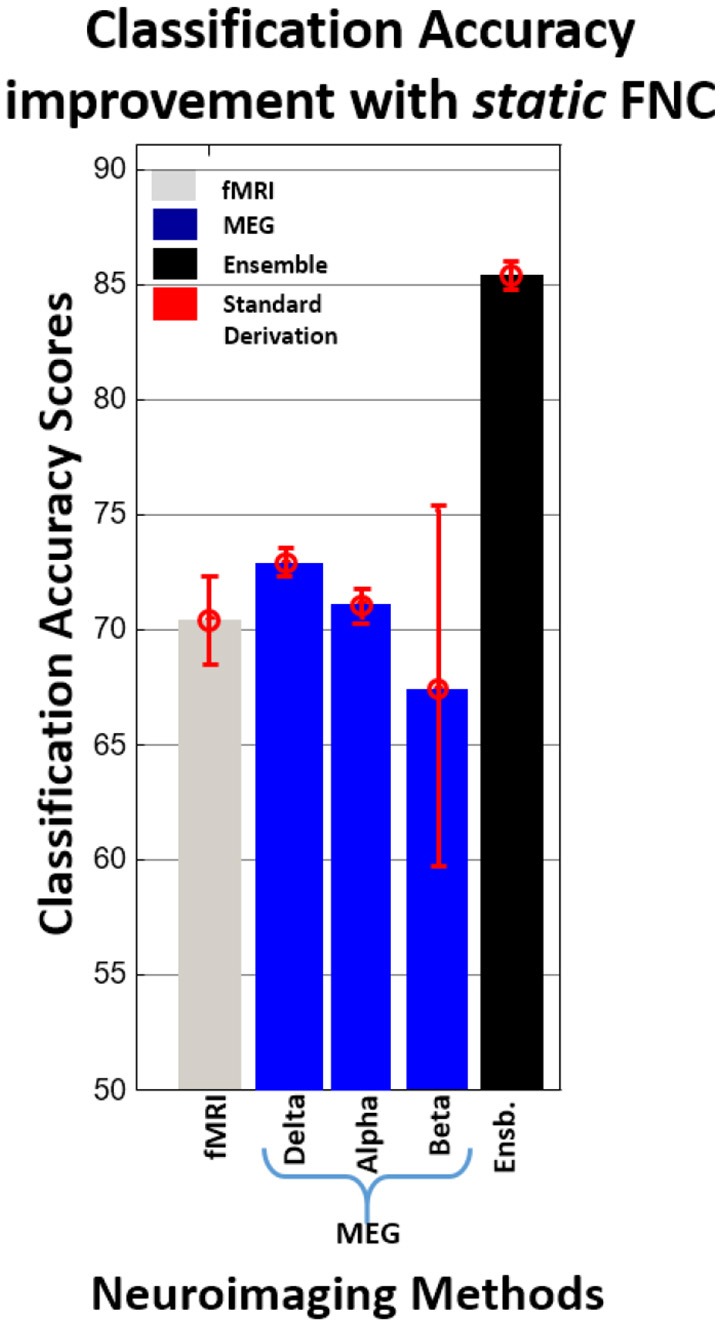
**Average classification accuracy improvement with static FNC**.

Combination of fMRI and MEG—Delta provided the best classification performance (81.69%) than that of other combinations. Figure 5 summarizes the average classification accuracy improvement with static FNC.

### Dynamic FNC classification

We evaluated the performance improvement of classification based on dynamic FNC and the combination of estimated networks from both MEG and fMRI methods. Our main focus was to extract reliable features from the *dynamic* FNC matrices as described in the “*Dynamic Functional Network Connectivity and Feature Selection*” section and combine these features (beta coefficients) from the training dataset to achieve the best classification results.

First, we used fMRI *dynamic* FNC matrices and MEG *dynamic* FNC matrices separately (for each frequency) for classification (see Table 6) then we combined fMRI (subject × time × FNC = 91 × 119 × 703) and MEG (frequency × subject × time × FNC = 5 × 91 × 270 × 496) dynamic FNC matrices as a data set for classification (see Table 6). We then compared results to show the improvement of combining beta coefficients from fMRI and MEG methods for classification.

**Table 6:** **Classification accuracy obtained from dynamic FNC for fMRI beta coefficients, MEG beta coefficients for each frequency, and combination of all MEG beta coefficients frequency bands by using majority voting method**.

**Acc%**	**fMRI**	**MEG**					
		**Delta (%)**	**Theta (%)**	**Alpha (%)**	**Beta (%)**	**Gamma (%)**	**Ensemble (%)**
NBC	82.42	71.43	53.85	65.93	71.43	51.65	65.93
nSVM	83.52	69.23	58.24	69.23	72.53	51.65	69.23
LDF	82.42	71.43	53.85	65.93	68.13	52.75	65.93
Average	82.79	70.70	55.31	67.03	70.70	52.02	67.03
(std)	(0.64)	(1.27)	(2.53)	(1.91)	(2.29)	(0.64)	(1.91)

Although, static FNC results showed that there are no significant group differences for MEG-gamma and MEG-theta frequency bands, we used all dynamic FNC MEG frequency bands to assess whether the property of dynamic FNC would capture significant transient differences not measureable with static FNC.

Table 6 provides the classification results that were obtained from beta coefficients of fMRI and MEG data for each frequency and combination of all MEG data frequency bands by using a majority voting method. Results show that the classification accuracy obtained from beta coefficients of fMRI data (82.79%, Average accuracy scores of classification algorithm) provides better classification performance than beta coefficients of MEG data for all frequency bands considered independently and for the combination of all MEG frequency bands, using a majority voting method. Comparison of MEG frequency bands shows that classification of beta and delta (70.7%) frequency performed better than other frequency bands and the combination of all MEG frequency bands. Similarly, FDR-corrected group differences of MEG-beta and MEG-delta frequency bands show more significant differences than other bands.

Table 7 summarizes the classification accuracy obtained from the combination of beta coefficients from fMRI data and MEG data for each frequency and the combination of all by using a majority voting method. Combination of features obtained from dynamic FNC of fMRI and MEG-Beta frequency provided better results (85.71%) than other frequency bands. The best performance is provided by the combination of all by using a majority voting method (87.91%).

**Table 7:** **Classification accuracy obtained from the dynamic FNC for the combination of fMRI data and MEG data for each frequency band and the combination of all by using majority voting method**.

**Acc %**	**fMRI**					**Ensemble (%)**
	**MEG Delta (%)**	**MEG Theta (%)**	**MEG Alpha (%)**	**MEG Beta (%)**	**MEG Gamma (%)**	
NBC	86.81	85.71	83.52	87.91	83.52	90.11
nSVM	84.62	81.32	82.42	85.71	82.42	87.91
LDF	83.52	83.52	82.42	83.52	82.42	85.71
Average	84.98	83.52	82.79	85.71	82.79	87.91
(std)	(1.67)	(2.20)	(0.64)	(2.20)	(0.64)	(2.20)

We repeated the clustering method by using different distance functions such as Euclidian, correlation, and cosine similarities. We did not find any performance differences.

**Figure 6:**
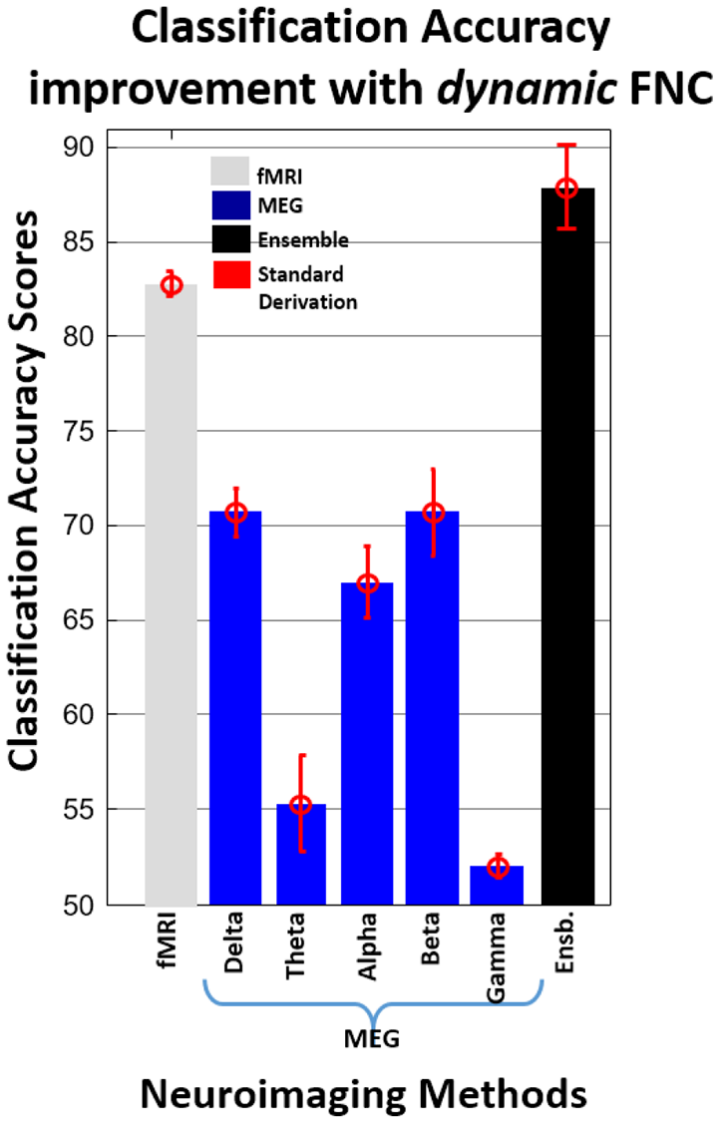
**Average improvement in classification accuracy with dynamic FNC**.

Figure 6 summarizes the average improvement in classification accuracy with dynamic FNC. fMRI showed better individual classification accuracy performance (82.79%) than all MEG frequency bands. Classification accuracy performance of the combination of beta coefficients from fMRI and individual MEG frequency bands are higher or equal to the best individual classification accuracy (fMRI). When considering the combination of beta coefficients from fMRI and MEG—Beta (Average accuracy performance of classification algorithms) provided the best classification performance (85.71%) relative to other combinations. Similar to static FNC, the best classification accuracy was achieved by the ensemble of fMRI and all MEG frequency bands.

## Discussion

In this study, we present an initial effort to investigate the classification performance of intrinsic connectivity networks from group spatial ICA of fMRI and MEG data by using FNC in a sample of SZs and HCs. Two types of FNC methods (static and dynamic) were used to examine our initial hypothesis that multimodal data (fMRI and MEG) improve the prediction of mental illness, such as schizophrenia, in individuals.

The results of this study support our initial hypothesis. FNC results suggest that the application of Sg-ICA to multimodal neuroimaging using MEG and fMRI provides important information about schizophrenia that represent fundamental characteristics of brain network connectivity that would have been missed otherwise. The combination of data from MEG and fMRI, collected on different days, also allows us to rule out multiple alternative explanations for the observed results, including scanner noise.

Some interesting network patterns are seen in the MEG (Delta, Alpha, and Beta frequency bands) FNC results (see Figure 4). For instance, we see hyperconnectivity between DMN and Frontal networks for SZ compared to HC (blue regions) while within the Frontal networks, HCs show hyperconnectivity compared to SZs (red, yellow, and orange regions). Also, we see very similar pattern (hyperconnectivity pattern of DMN and Frontal network) between Frontal networks and Auditory network. Our results support the previous studies of schizophrenia that similarly showed hyperconnectivity patterns for the same functional networks (DMN-Frontal network and Frontal network—Auditory network) between HCs and SZs (Jafri et al., 2008; Skudlarski et al., 2010).

In particular, we want to point out MEG within-frequency band FNC (see Figure 2) which showed multiple group differences in inter-regional connectivity in the beta (16–29 Hz) range also demonstrated high variability observed relative to the other bands, particularly in the frontal-cerebellar, frontal-DMN, and frontal-auditory networks. Nearly all FNC group differences in the beta range suggested hyperconnectivity in patients. Beta band has previously been implicated in long-range cortical synchrony (von Stein et al., 1999; Tallon-Baudry et al., 2004; Thatcher et al., 2008), notably in visual processing (Sehatpour et al., 2008) and working memory (Piantoni et al., 2015) networks observed in the present data. Consistent with the present study, research in schizophrenia has indicated abnormal synchronization in the beta band. Synchrony between hippocampi, regions with particular relevance for schizophrenia (Hanlon et al., 2011, 2012), has also been linked to altered beta band activity (Lee et al., 2014).

For testing our hypothesis with static and dynamic FNC data, we used fMRI data and all MEG frequency bands. MEG outperformed fMRI with static FNC, fMRI outperformed MEG with dynamic FNC but combined fMRI + MEG outperformed either modality separately for both static and dynamic FNC. It is important to note that the MEG data were down-sampled to 1 Hz similar to Brookes et al. thereby limiting the MEG dynamics. This is discussed in more detail in the limitations of the study below. Classification accuracy of combined fMRI+MEG was improved up to 15% relative to only the fMRI method and up to 12.45% relative to only the MEG method by using static FNC and up to 5.12% relative to only the fMRI method and up to 17.21% relative to only the MEG method by using dynamic FNC. Also, the present study is consistent with previous results (Bridwell and Calhoun, 2014; Ingalhalikar et al., 2014; Silva et al., 2014; Sui et al., 2014; Cetin et al., 2015b) suggesting that by increasing the variety of data types, the classification accuracy is improved.

Comparison of dynamic and static FNC results for fMRI showed that dynamic FNC pipeline provide better individual prediction score (82.79%) for schizophrenia at the single-subject level then statistic FNC (70.33%). These finding are consistent with Rashid et al. (2015) where they compare individual prediction performance of static and dynamic FNC for schizophrenia, bipolar disorder, and healthy control. Also, individual prediction performance of ensemble dynamic FNC (87.91%) is higher than ensemble static FNC (85.35%). Comparison of dynamic and static FNC results for MEG-Beta band showed similar results with fMRI (67% for static FNC and 70.7% for dynamic FNC). On the other hand, individual prediction performance of static FNC (MEG-Delta 72.9%, MEG-Alpha 71%) is higher than dynamic FNC (MEG-Delta 70.7%, MEG-Alpha 67.03%) for MEG-Alpha and MEG-Beta bands.

Classification results obtained from static FNC show that fMRI and MEG (alpha, beta, and delta frequency bands) have relatively similar classification power for SZs and HCs. MEG-beta frequency provided the highest number of significant features which supports previous studies (Brookes et al., 2011b; Luckhoo et al., 2012; Hall et al., 2013; Nugent et al., 2015). Although, we achieved a high number of significant features from MEG-beta frequency, performance on classification accuracy is not significantly different from MEG-alpha and MEG-delta frequency bands. For dynamic FNC, classification accuracy scores of fMRI were significantly higher than MEG frequency bands. Classification accuracy scores for MEG frequency bands were similar except for MEG-gamma and MEG-theta frequency bands. These two frequency bands showed significantly lower classification accuracy scores than the other frequency bands. These results support our decision of not including these two frequency bands with static FNC in this study.

We consider the current results a first step in developing more sophisticated classification frameworks for mental disorders by using a group ICA approach with FNC for fMRI and MEG methods. The current study had some limitations such as (1) Anatomical labeling of functional regions comes with limitations as it is hard to provide an exhaustive list of all regions included within a component, thus we label based primarily on the peak region. (2) The higher temporal resolution of MEG was not the focus of this study. It was therefore limited by using the same down sampling rate (1 Hz) for all the MEG frequency bands and informative dynamic information may have been lost in the process, (3) for dynamic FNC analysis, the same window length (default setting of GICA for fMRI) was used for fMRI and MEG data. These limitations may cause the loss of information for connectivity analysis and poor classification accuracy, especially for MEG method. For future studies, a higher classification accuracy score may be achieved by using an optimal down sampling rate for each MEG frequency and an optimal window size for dynamic FNC.

Another limitation is that the leave one out method is applied after a part of the parameter extraction (in sg-ICA process). That could potentially bias the accuracy estimate. In order to address this issue, we created a sub set of all data sets (Since computation of all steps would take years to do with all data sets) with the same distribution of original data sets and repeated through all the processes with created dataset. Results (ICA components) did not show any significant differences (in terms of parameter extraction) to those we reported in the paper and do not show evidence of bias. In our future work, we will use the benefits of a super computer to apply parameter extraction (in sg-ICA process) before separation of training and testing data.

## Conclusion

Innovative feature extraction approaches from group level networks obtained with Sg-ICA using both MEG and fMRI data sets could be very effective for diagnosis of mental disorders. Our results provide evidence that the combination of fMRI and MEG modalities provides important information for classification that is missed by using only one modality. This suggests that the combination of these two methods provides valuable information that captures fundamental characteristics of brain network connectivity in schizophrenia. These results may help guide the design of an objective biological marker for the early diagnosis of mental disorders such as schizophrenia.

## Author contributions

All coauthors contributed to this study: MC, JH, BR, OA, JMS, JS, JC, AM, CA, JB, VC.

## Funding

This work was partially supported by National Institutes of Health grant R01EB006841, National Sciences Foundation grant 1016619, the Centers of Biomedical Research Excellence (COBRE) grant P20RR021938/P20GM103472.

### Conflict of interest statement

The authors declare that the research was conducted in the absence of any commercial or financial relationships that could be construed as a potential conflict of interest. The reviewer MJR and handling Editor declared their shared affiliation, and the handling Editor states that the process nevertheless met the standards of a fair and objective review.
